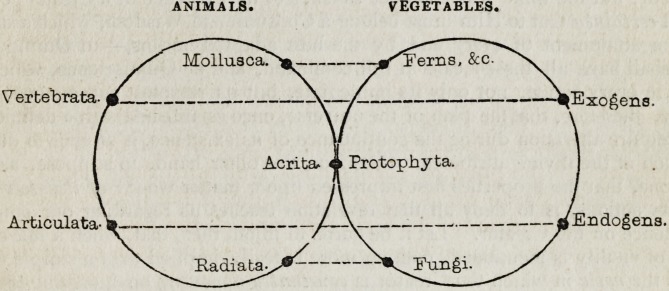# Principles of General and Comparative Physiology, Intended as an Introduction to the Study of Human Physiology, and as a Guide to the Philosophical Pursuit of Natural History

**Published:** 1839-01

**Authors:** 


### ART. IX.

*Principles of General and Comparative Physiology, intended as an Introduction to the Study of Human Physiology, and as a Guide to the Philosophical Pursuit of Natural History.* By WILLIAM B. CARPENTER, Member of the Royal College of Surgeons, London; Lecturer on Forensic Medicine in the Bristol Medical School, &c. &c. With 240 Figures in Copper and Wood.—*London*, 1839. 8vo. pp. 480.

THE work before us has equalled our most sanguine expectations. This would be recognized as high praise, were we to relate all that our knowledge of the mental qualities of the author, and of the attainments which have fitted him for his undertaking, had led us to look for. But this we shall, for various reasons,—some of which our attentive readers will readily understand,—omit on the present occasion; satisfied that, whoever shall carefully peruse Mr. Carpenter's book, will not fail to discover as much as we had ventured to anticipate. The excellencies of many standard works on physiology are shared in no slight degree by this production; for, if we do not mistake, it combines much of the close reasoning of Alison, and of the comprehensive philosophical spirit of Fletcher, with the richness in details and the accuracy of statement which characterize the respective writings of Tiedemann and Müller. But we forbear saying all that we feel on this subject, and hasten to give our readers some idea of the plan of the work; the nature of which, however, renders it impossible for us to offer, within the limits by which we are confined, a full analytical account of it.

The work is divided into two books; the first comprising General Physiology, the second Special and Comparative Physiology: but these are preceded by an introduction of considerable length, and of corresponding importance, presenting a summary of the characters of organized structure, and a general view of the vegetable and animal kingdoms. The most characteristic feature of the whole work is the attention paid to vegetable physiology; and we are acquainted with no other treatise which exhibits the analogical bearings of modern discoveries in this most interesting science on the physiology of animals, with anything like the same extent and precision, or, at all events, in so systematic a form.

The peculiarities in the mechanical arrangement of organized bodies, as contrasted with inorganic, are a definite form, a determinate size, an individuality, a consistence tending to softness, and a peculiar chemical composition: upon these, although familiar points, the author offers some very interesting remarks, noticing particularly the indistinctness of these characters in the lower degrees of organization. Thus, with regard to the first, its diagnostic value is lessened in the inferior cellular plants and in the lowest animals, as sponges and polypifera, which are somewhat deficient in definitiveness of form; "and there is reason to believe that, among these, the same germ may assume a variety of distinct forms according to the circumstances under which it is developed, just as the same mineral substance may present itself under a diversity of crystalline shapes." (p. 12.) *Size*, again, is much less limited in the simpler organisms: witness the enormous length to which sea-weed may attain, and the almost immeasurable extent of a coralline growth. The third character, or *individuality*, lessens materially as we descend the scale: thus, many plants and animals, instead of consisting of parts all subservient to the whole, are assemblages of independent members, which are repeated, as it were, throughout the fabric. The gradation of this character is thus happily expressed: "The individuality of a mineral substance resides in each molecule; that of a plant, or inferior animal, in each member; and that of one of the higher animals in the sum of all the organs." *Softness* is a striking distinctive character of the organic kingdom, as the opposite quality is of the mineral; but in the lowest animals we find a great predominance of earthy materials,—in the corallines, for instance. Correspondingly, the parts of vegetables and animals which exhibit least activity of vital processes, such as the woody fibre and osseous tissue, have the greatest hardness; while the nervous matter, which is furthest removed from inorganic substance, is the softest and most decomposable. The remarks on the chemical composition of organic bodies deserve particular attention, as showing the incorrectness of the common assumption, that the affinities which hold the elements together during life are different in their nature from those which dissever them after death. Reasons also are adduced for doubting whether the organic proximate principles have really such arrangements of their elements as are called ternary and quaternary.

We have next a condensed description of the elementary tissues in plants and animals, in which we can only take notice of one or two well-marked analogies. After describing the spiral vessels of plants, the author observes :

"A very curious analogy to this structure is exhibited in the tracheæ, or air-tubes, of insects, which ramify by minute subdivisions through the whole of their bodies. These tubes are formed, like the spiral vessels of plants, of an external membrane distended by spiral fibre, which is coiled with the most beautiful regularity; the principal difference in these two structures being that the air-tubes of plants are closed vessels, and that their gaseous contents find their way through the delicate membrane which composes them by the capability of permeation, which will be subsequently described : while the tracheal system of insects exhibits the most beautiful and minute ramifications, formed by the subdivision of its principal trunks, which communicate directly with the atmosphere." (p. 25.)

A little further on he shows how the *dotted duct* is a degeneration from the type of a spiral vessel, and then instances a similar departure from the original type in the irregular patches of cartilage distributed over the bronchial ramifications of the tracheæ. Between the adipose tissue of animals and those parts of the cellular membrane of vegetables which contain oil or gummy matter, stored up for the future nutrition of the organism, there is a striking resemblance. In both kingdoms, again, we find that the cellular, or most simple tissue, is most largely employed in the fabric of the higher organisms, and composes the entire bulk of the lower forms. In this respect the translucent globular tissue which clothes the skeletons of the porifera corresponds well with the "loosely aggregated gelatinous tissue which constitutes some of the lowest plants."

From the section devoted to a cursory view of the vegetable kingdom, which is intended to exhibit the principles of classification connecting the several groups, and to furnish as much natural history as may be requisite for understanding the subsequent departments of the treatise, we extract the following table.

"70. The affinities of the principal divisions of the vegetable kingdom may be generally expressed in the following manner:

EXOGENS. ................ENDOGENS.

Gymnospermæ. Rhizantheæ.

*Ferns, &c.*—*Protophyta.*—*Fungi.*

ACROGENS.

Starting from the simplest algæ and lichens, we may pass, on one side, through the hepaticæ and mosses, to the ferns, the highest among the acrogens or cryptogamia. From mosses and ferns the transition is easy to exogens, through lycopodiaceæ and gymnospermæ. Exogens and endogens have many connecting links; and from the latter group the return to the fungi is direct by the rhizantheæ; whilst the simplest forms of the fungi bring us back again to the protophyta." (p. 66.)

The connexions between the great divisions of the animal kingdom are traced in a similar manner. Thus, starting from the simplest forms of the acrita, we pass easily by the polypes to the tunicata, or lowest tribe of the mollusca; and from these by the cephalopoda to the vertebrata. From the vertebrata there is a ready transition to the articulata through the suctorial fishes (which have so strong a resemblance to leeches), or, as it regards *animality* of function, by the insects. The articulata are manifestly connected with the radiata, by the cirrhopoda of the former, and the holothuria and siponculus of the latter. We return from the radiata to the acrita "by numerous links of transition, such as the group of acalephæ, the actiniæ (which approach so near to some of the star-fish), and others. The circle of affinities may therefore be expressed in the following manner:

*Cephalopoda.* VERTEBRATA. ARTICULATA. *Holothuridæ.*

MOLLUSCA. ACRITA. RADIATA.

Again, the respective positions of the principal groups in the animal and vegetable kingdoms are set forth in a third and ingeniously devised table, which we cannot forbear introducing, for the sake of completing the view.

ANIMALS. VEGETABLES.

The section which treats of the Symmetry of organized Structures contains some important observations; but, having already devoted as much space as could be well spared to the introduction, we must pass on to the General Physiology, the first chapter of which treats of the *Nature and Causes of Vital Actions.*

The doctrine respecting Life now, it is to be hoped, prevailing among the best informed upon the subject, is ably set forth, and strengthened by additional facts and considerations. Regarding life in the abstract as synonymous with vital action, or, in any one living being, as the aggregate of phenomena by which that being is characterized, the author shows that, instead of looking for its cause in an imaginary vital principle, or organic agent, presumed to exist for the sake of explaining the phenomena, we ought to study the properties which organized structure enjoys, and the agents which produce their manifestation. Some observations are made in refutation of the doctrine of a vital principle, and we do not think them supererogatory; for, although the hypothesis could have hardly been expected to survive the fine scientific thrusts of Dr. Prichard's classic weapon, or the strokes of Dr. Fletcher's more truculent blade, it seems even yet not quite extinct. Mr. Carpenter argues on the superfluousness of a controlling or presiding agent, intermediate to the will of the Deity and the phenomena of vital actions, when the latter can be reasonably assigned to the reciprocal relation between the properties which belong to organized structures and the stimuli which excite them. No agent can be required to adjust and regulate the actions which ensue from this mutual adaptation; since they are, like all other phenomena in the universe, under the control of laws inseparable from their very existence. The following passage will serve as a specimen of the manner in which the author treats this part of his subject:

"147. The term *law* of nature, as already employed, expresses the *conditions of action* of the properties of matter. The divine Creator of the universe 'has, by creating his materials, endued with certain fixed qualities and powers, impressed them in their origin with the *spirit*, not the *letter* of his law, and made all their subsequent combinations and relations inevitable consequences of this first impression.' In other words, the unchangeableness of His nature is manifested by his continued oction in the material creation, according to the same plan by which He at first adjusted the relations of its parts. Our belief in the uniformity of nature, which leads us to seek for a common cause when a number of similar phenomena are presented to our observation, is based not only upon experience, but upon the conviction which every believer in the existence of the Deity feels of His immutability. If it were otherwise, we should be led by *analogy* only to infer the existence of law and order where none is evident; but the mind which is once satisfied of the existence of a Creator possesses a moral *certainty* that to Him must belong a Consummate Wisdom, which shall contrive the attainment of every end by the best adapted means,—an Omnipotence, which shall have all these means at full command, and an Omniscience, which shall foresee in every action, not only its immediate, but its remotest consequences. To imagine, therefore, that the plan of the universe, once established with a definite end, could require alteration during the continuance of its existence, is at once to deny the perfection of the divine attributes; whilst, on the other hand, to suppose, as some have done, that the properties first impressed upom matter would *of themselves* continue its actions, is to deny all that revelation teaches us regarding our continued dependence on the Creator. Let it be borne in mind, then, that, when a *law* of physics or of vitality is mentioned, nothing more is really implied than a simple expression of the *mode* in which the Creator is *constantly* operating on inorganic matter or on organized structures." (p. 134.)

It excited our surprise, when reading the physiology of Müller, to find him clinging to a notion which it is high time should become obsolete. His "organic force" corresponds in most respects to the "vital principle." "Life," he says, "is not simply the result of the harmony and reciprocal action of these parts; but is first manifested in a principle or imponderable matter which is in action in the substance of the germ, enters into the composition of the matter of this germ, and imparts to organic combinations properties which cease at death."* We can only account for the retention of such an opinion in the mind of one who generally thinks so accurately, by the strong intuitive tendency, inherent in every one, to suspect the operation of hidden agents; an instinct, which is, for the most part, beneficial by stimulating to a more minute investigation of pheno-

* Müller's Physiology, by Baly, p. 28.

mena, but which may be excessive by preventing us from ever recognizing the adequacy of causes for any given event. Dr. Thomas Brown has finely shown the effect of this tendency in the popular notion of *power*.

Vital properties and vital actions are legitimate objects of enquiry; the former being, like all other properties, the expressions of our experience respecting the bodies of which they are predicated. A property is the capability of acting, or susceptibility of being acted upon, in a certain manner, under certain circumstances; but it can only be ascertained to exist in a substance by means of the actions which it either engenders or suffers. A vital action is one that is only observed in an organized body under the operation of certain stimuli; and, correlatively, the capability of exhibiting such an action is called a vital property. The question then occurs, by what means have organic bodies become possessed of such properties? In any other case it would be deemed sufficient to look to the constitution of the substance, and the circumstances in which it is placed; but, in the present instance, physiologists have thought proper to suppose the properties to be communicated *after* the substance has been organized, as if properties were entities susceptible of addition and subtraction. This mistake has, we apprehend, originated in part in the erroneous confounding of properties with *causes*. Vitality is not a cause of vital action, but the character of a body which displays such action; the cause must be sought in the events which have preceded the constitution of the body itself. A substance cannot be endowed with new properties without undergoing some change in its own condition, of which altered state these properties are the necessary attendants. The author adverts to a supposed illustration of this communication of properties to matter, which did not before possess them, in the case of iron, on which magnetic properties have been superinduced: but even in this instance, as he justly remarks, the magnetic properties are developed only because the conditions of the metal itself are altered; and, in like manner, "the act of organization developes vital powers in the tissues which it constructs."

We have both seen and heard it maintained that life cannot be the result of organization, because the existence of the latter implies a previous action of the former. It is true that we only know organization as the result of vital action; but not less true is it that we know nothing of vital action, as separate from organized structure. Trace the living being back to its germ; that germ is the product of vital action, but the action is in the tissue of the parent; and, in pursuing the backward course, we arrive at the creation of species. Shall we believe that God created a vital principle, or an organic agent, and then set it to organize the body? Surely it is, to say the least, more natural to conceive that the organized species, whether a germ or a mature being, was, *quà* organized, vital, or capable of exhibiting life as soon as the appropriate stimuli should be applied. But while we believe that life is thus begotten, as it were, of external agents upon organized structure, we must never lose sight of how much is implied in the latter.

The dependence of the vital properties on the structure, Mr. Carpenter enforces by a consideration of the nature of death; showing that when integrity of the organization is maintained by the continuance of its vital action (particularly nutrition), the change of structure consequent on the cessation of the action necessarily involves the loss of vitality. *Molecular* Death, so designated and distinguished by Dr. Symonds from *systemic* or *somatic* death,* may in most cases be said to consist in the cessation of vital action in the part, because the latter, as we have just observed, necessarily deprives it of its vital properties by producing its disorganization. But there are exceptions to this statement in the case of seeds which retain their vitality for an almost indefinite period, though it cannot be said that they are the subjects of vital action; and also in certain animals of comparatively simple organization, in which life may be suspended for a considerable time without deprivation of vitality. Thus Mr. C., and others, have found that the wheel-animalcule may be reduced by desiccation to a state in which vital action not only appears extinct, but which would seem quite incompatible with its continuance; yet, on the restoration of moisture, life returns. In both these cases the structure is not readily susceptible of decomposition from the operation of ordinary external agents; and, consequently, its integrity is not dependent on the continuance of vital action; and being unimpaired in organization it retains its vital properties.

The author notices those actions and properties in organized tissues which are of a purely physical nature, such as elasticity, and endosmose; and then adduces strong reasons for believing that certain affinities usually considered vital, or not reducible to the ordinary laws of chemical attraction, are really identical with the latter, the *conditions* under which they act being so complicated that we are at present unable to imitate them. The affinities alluded to, we need scarcely say, are those by which the organic proximate principles, such as sugar, gum, albumen, &c. are formed from the nutriment introduced into the living system. Could we place these elements in the same mutual position and under the same agencies, as in the organized being; could we, in fact, adjust the temperature, the moisture, the electricity, the light, and the heterogeneous atoms to be acted upon, just as they are employed in the laboratory of nature; we entertain not the slightest doubt that the ensuing actions would be the same, and that they would be found to be governed by the same laws of chemistry as regulate even inorganic processes. Independently of this consideration, it must be remembered that the laws of inorganic chemistry are as yet so imperfectly known, that it would be presumptuous to pronounce any molecular combination to be beyond their influence or control. The following remarks should be well weighed by those who are too prone to infer a new law or power from facts, apparently irreconcilable, with what knowledge they possess.

"Those who have attended to the progress of chemical science during the last few years can scarcely hesitate in the belief that we as yet know little of the laws which govern the changes in the constitntion of bodies, compared with what future discoveries will reveal to us. Many phenomena of inorganic chemistry, which can now be readily explained, would have been regarded, within a very recent period, as quite incomprehensible. Would it have been thought possible, for example, by a chemist thirty years ago, that the same substance should act the part of an *acid* in one case, and of a *base* in another?—that *water* should be possessed of such properties?—or, still more, that *muriatic acid* should act as the *base* or electro-positive ingredient in

* Cyclopædia of Anatomy; Art. *Death.*

combination with the chloride of platinum? These facts would have appeared to a chemist, at the commencement of the present century, totally inconsistent with what he knew of chemical action; but they are now readily comprehended, as results of laws which, being higher and more general than those previously known, include facts that at first sight appeared inconsistent with them. Unless, therefore, a distinct set of laws could be established, regulating vital affinities—which has not been accomplished or even attempted—we are scarcely justified in assuming that these laws may not be accordant with those which we resognize elsewhere." (p. 147.)

After deducting those actions which are unequivocally physical, as well as the more questionable processes of organic chemistry, the author allows that there remains a vast chaos, which must be regarded as *essentially* vital; by which we presume he means that they are not only confined to, but that it is impossible that they should occur in any but living organized structures, since they require properties which are not to be met with in any other substances; while, on the other hand, the formation of the proximate principles may demand only the play of the general properties of matter under peculiar limiting conditions, which it may not be beyond the reach of art to imitate. As instances of the purely vital actions, the author adduces the conversion of organic compounds into the substance of living tissues. We cannot help noticing that in this place he uses a phrase inconsistent with the views elsewhere stated. Thus, he speaks of the compounds "being converted into organized tissues, and *endowed with properties*," &c. and, again, of a process concerned "in assimilating, *organizing, and communicating vital properties* to nutritious matter." Now, if the view which he has taken, and which we have supported, of the connexion between organization and vital properties, was well-founded, it is incorrect to use such phrases as those which we have marked in italics, since the organizing is, in fact, the same thing as communicating vital properties, that is, forming a structure the essential character of which is the possession of such properties. We readily admit that such slips of expression are very pardonable in writing on such a subject, but they are, in the present instance, in too close a juxta-position with the argument which demonstrates their impropriety, to allow of our passing them entirely *sub silentio.*

The second chapter treats of the *Vital Stimuli.* These are principally those external agents which, if they do not act immediately upon the properties of the higher organisms, are essential to the elaboration of the fluid which at once supplies the materials of growth and secretion, and stimulates the tissues to their proper functions. The influence of nervous matter upon the irritability of muscular fibre is an instance of an *internal* stimulant. The agents which excite the functions of relation, such as the stimuli of sensation, and of such muscular actions as do not directly conduce to the maintenance of life, Mr. C. is unwilling to include under vital stimuli; but his objections are not, in our opinion, altogether valid. Sensibility and muscular contractility, whatever be the ends to which they may be made subservient, are unquestionably vital properties, the term being used in the sense which the author generally follows, viz. peculiar to organized structure, and therefore any influence which calls them into exercise may be fairly designated a vital stimulus. The author notices *sneezing* produced by irritation of the nostril, as an action excited by a stimulus not deserving the character *vital,* because the operation is but very remotely connected with the continuance of life. Yet this action appears to us rather more important, since its object is that of forcing a sudden and impetuous current of air through the nostrils, in order to expel any substance obstructing those passages; being precisely of the same nature as coughing, the object of which is to clear impediments from the bronchi and trachea.

Food and air, as vital stimuli, are considered under Absorption and Respiration. The present chapter is devoted to the influence of *Heat*, *Light*, and *Electricity*, and contains a great variety of interesting facts, many of which were new to us. What extremes of temperature are required by different vegetables, is well illustrated by the protococcus nivalis, or *red snow*, which flourishes only on the frozen tracts of the Arctic regions; and by certain confervæ which have been found growing in boiling springs. The influence of heat in destroying the vitality of seeds is physical. Water at 144° causes the rupture of the vesicles of starch, and being thus disorganized the seeds cannot germinate; yet these bodies will bear exposure to a degree of cold equal to that of frozen mercury. Many animals are enabled to bear extremes of temperature by their power of modifying it; but in some which have not this power, it is found that a high degree of heat is compatible with vital action: thus fishes exist in the thermal springs of Barbary, at a temperature of 172°. The different degrees of *light* required by different vegetables, are exemplified in the cryptogamic plants which grow on the northern sides of trees and towers, while other plants can only flourish under the powerful rays of a tropical sun. The influence of light in retarding germination is very interesting, because "to the chemical process which this requires, light would be decidedly opposed, tending as it does to *fix* carbon in the system, instead of favouring its liberation." Mirbel relates a striking fact in proof of the influence of light on the development of vegetable organs.

"He found that up to a certain period of the growth of the little *gemmæ* of the Marchantia polymorpha, it appeared indifferent which side was uppermost; for that, on the surface of the foliaceous expansion, exposed to the light, *stomata* would always be formed, while, from the under surface, roots would be protruded. After the tendency to the formation of these organs had once been given, however, by a sufficiently protracted influence of light above, and of moisture beneath, it was in vain to attempt to alter it; for if the surfaces were then inverted, they would be restored to their original position by the twisting growth of the plant." (p. 157.)

We have not space for pursuing further these and other interesting details regarding the vital stimuli; else we should have been glad to have quoted what is said of the capability which certain salt-water and fresh-water fishes possess of bearing a change in their medium, if this is effected gradually, while others are soon disordered and die when removed from salt to fresh water, or from this to that. Some of the littoral mollusca it seems are fond of variety, for they fix themselves to rocks at the mouths of rivers, where with the alternation of the tide they enjoy corresponding changes in the quality of their element.

The *Laws of Organic Development* are explained in the third chapter, under the several heads of Unity of Composition—Progressive Development—Eccentric Development—Balancing of Organs—Harmony of Forms. This high department of physiology is managed with the same ability which pervades the other parts of the work, both in the announcement of the laws, and in their exemplification; but on the first, or the Unity of Composition, there appears to us to be some little obscurity or confusion of statement; for while it is maintained, and justly, that no fundamental unity of structure between the various classes of animated beings can be founded upon similarity of external form, or even upon analogy of function, yet the author appears to determine the community of structure by what he calls "the essential characters," and "the real analogies," which seem to have reference to a structure fitted for a particular action. Thus, he notices the diversity of form in the respiratory apparatus of different animals, and shows how they agree in the possession of this character, that "a membrane should be in contact with air on one side, and with fluid on the other." Surely this "essential character," has reference to the function, that is, the action between the air and the fluid; if not, it belongs also to the tegumentary and some parts of the digestive apparatus. But how apply this to the swimming bladder of the fish which is a rudimentary lung? There is a "real analogy" (being purely structural) between these parts, but no community of function. It does not, to our apprehension, remove the difficulty to say that a portion of the respiratory apparatus is "modified to adapt it to the condition of the structure at large." Indeed, with reference to this very instance, Mr. C. observes that it proves the necessity of disregarding function in investigations of this nature; a remark which we know not how to reconcile with what was said of the essential character of the various forms of respiratory apparatus. The truth appears to be, that a character is essential or not, and an analogy real or not, according to the view which, for the time being, we are taking of a number of organs. If it be for the purpose of finding a community of *function*, then the essential character must be the structure which is needed by the function, and which is a true analogical character. But if we are viewing them with reference to their unity of *structure*, the analogies we look for must be sought in the mere anatomical elements; thus the elements which compose the tympanal bones of the fish are the *ossicula auditûs* in higher organisms, and the scrotum of the human male is the analogue of the female nymphæ. The determination of these structural analogies can be only attained by profound and extensive zootomy, conducted through the various metamorphoses of organs, in different classes, or in the same individual at different periods. The theoretical result of such researches is that the great divisions of living beings have an identity of structural elements, which variously compiled, and developed in ever-varying degrees, produce organs whose forms and functions are infinitely diversified, according to the plan on which the class or species is formed. It strikes us, then, that the author has applied to the law of unity of structure, or identity of elements, illustrations which belong to "unity of function." To set forth the difference between a functional and a structural analogy in a still clearer light, we may take one or two examples. Thus the lung of the batrachia and the gills of the fish display a functional unity, but the former has its structural representative in the air-bladder of the fish, while the anatomical elements corresponding to the gills of the latter will be found in a rudimentary or atrophied state. Again, the pectoral fin of a fish, and the wing of a bird, have no analogy of function, but their anatomical elements are the same, though developed in different proportions; and, on the other hand, the wing of a bird, and of an insect, share a character of analogy as close in point of function as any two specimens of respiratory organs, namely, a structure which, by its expansion, lightness, and mobility, may serve for supporting or impelling the animal in air; but the anatomical elements of the two are utterly different; for the one is moulded out of the anterior extremity of a mammal, the other of structural elements which correspond to the respiratory apparatus in earlier periods of development. That the author has in his eye rather a unity of function than of *composition*, we should gather from the following statement: "Throughout the whole animated Creation, then, the essential character of the organs which all possess in common, remains the same; whilst the mode in which that character is manifested varies with the general plan upon which the being is constructed." (p. 168.) The organs hinted at are organs in the strict sense of the word—*instrumental structures*—and their essential character is that part of their structure which is essential to their instrumentality, though it may be embodied in very different forms. The statement is, we consider, perfectly true, and it is thus interestingly illustrated.

"Thus, in the lowest plants, as in the embryo-state of animals, the whole surface is modified for absorption of nutrient fluid; and the only change in the character of this absorbent surface in the higher vegetables consists in its restriction to a certain part of the structure, the root, which is developed so as to bring it into most advantageous employment. In animals, a change of a different character has become necessary to adapt the function to the conditions of their being; and we find the absorbent points distributed not upon the external surface, but upon an inversion of it, adapted to retain and prepare the food. Still the same fundamental unity exists; and the spongiole of the vascular plant, and the origin of the absorbent vessel in the animal, have precisely the same essential character with the membrane which constitutes the general surface of the Sea-weed or Red Snow. The advance from the lowest to the highest form in each kingdom is extremely gradual; and there are links of connexion between the two principal modifications of the structure, a plant exhibiting something like the digestive cavity and absorbent system of the animal, and certain animal forms absorbing from their general surface like the lowest plants." (p. 168.)

The remarks on Progressive Development are at once sound and lucidly expressed. The gradual concentration, or specialization, in the higher system of the diffuse organs which prevail in the lower is thus stated by Von Bär. "A heterogeneous or special structure arises out of one more homogeneous or general; and this by a gradual change." Another law somewhat restrictive of this has been discovered by Mr. C., and is thus expressed. "In cases where the different functions are highly specialized, the general structure retains, more or less, the primitive community of function which originally characterized it." Thus, though the absorbent function is highly specialized in the more complex organisms, yet every part of their surface still exhibits the primitive function, by its capability of admitting the passage of fluids into the interior of the system. But our limited space obliges us to take leave of this interesting subject. We can only remark, in passing, that we agree with the author in regarding Cuvier's "harmony of forms" as the result of other laws of development. "It is evident that if it were deficient, the race must speedily become extinct, the conditions of its existence being no longer fulfilled; these conditions being, for the whole organism, what the vital stimuli already described are for its individual properties." (p. 176.)

Chapter IV. contains a *General View of the Functions* and their mutual relations. First, we find pointed out the antagonism between the functions which maintain individual life and those which continue the species; next, the distinction between those of organic, and of animal life; and then a rapid view is taken of the characters and the connexion of the several functions which are treated of in detail in the special physiology,—viz. Absorption, Digestion, Circulation, Interstitial Absorption, Nutrition, Respiration, Secretion, Reproduction, Muscular Contraction, and the Functions of the Nervous System. The facts adduced in proof of an antagonism between nutrition and reproduction are highly interesting. For instance, while individual algæ attain an enormous size, (some species having a length of many hundred feet), their fructifying system is often obscure. On the other hand, the Fungi seem to consist of little else than reproductive organs, and after maturing their germs, the end of their being seems accomplished, for they then die. The same relation holds good in the flowering plants; and hence, in order to obtain fine fruit, that is, to give great development to the generative system, the gardener restrains the luxuriant growth of the plant, by pruning, or "limits the supply of food by trenching round the roots." The animal kingdom furnishes illustrations of the same antagonism. "Thus, in the larva condition of the insect, the assimilation of food, and the increase of its bulk, seem the sole objects of its existence." The imago or perfect insect, on the other hand, lives only to reproduce, and often dies without having taken food. In the young human subject, the generative system is dormant, while nutrition is in its greatest activity; and in adults, as Miss Martineau well knows, reproduction is apt to go on in an inverse ratio with the means of subsistence.

The author gives excellent reasons for withholding assent to the notion that "nervous agency" is essential to nutrition and secretion, though they are readily influenced by it. In vegetables these processes are highly complex and elaborate, and yet no trace of a nervous system has been discovered in this class of beings, nor indeed in the lowest animals, while "in the higher classes we find such an apparatus developed, just in proportion as the necessity arises, from the complication and specialization of the organic functions, for their being harmonized and kept in sympathy with each other and with the conditions of the animal system, by some mode of communication more certain and direct than that afforded by the circulating apparatus, which is their only bond of union in plants." (p. 184.)

It would be quite beyond the compass of this article to offer an abstract of the special and comparative physiology; but desirous of enabling our readers to form some estimate of the manner in which this department is executed, we shall, instead of culling from the different chapters facts and arguments which would be necessarily unconnected, prefer the plan of following the author through his treatment of a single function. The circulation will serve this purpose, conveniently to ourselves, and favorably but not *partially* to the author.

In the simplest organisms, both animal and vegetable, every part of the surface absorbs the nutrient fluid with which they are surrounded, and consequently they have no need, as in more complex forms, of an apparatus for conveying to the different parts a fluid ingested by a particular apparatus. Correspondently with this fact we find that the development of the vascular system is, in all classes of living beings, "proportional to the degree of limitation of the power of absorption, by which the parts imbibing aliment are insulated from those requiring supplies." But besides the conveyance of nutriment to the tissues, and of supplying a constant stimulus to their appropriate actions, the circulation fulfils the object of carrying the alimentary fluid to that part of the system in which it is to be subjected to the influence of the atmosphere.

The circulation in vegetables is first described. The first approach to a circulating apparatus (altogether wanting in the algæ) may be traced in the elongated cells which we find in the stems of the lichens. In this tribe, as "the power of absorption is usually restricted to the side least exposed to the light, more capability of diffusing the nutrient fluid is required; and it appears that when the absorbent surface is placed in water, the liquid is transmitted in the course of the elongated cells to the whole plant." In the higher fungi a distinct conveyance of fluid takes place from the radical fibres, through the elongated cells of the stem and the intercellular spaces, to the expanded summit of the plant. In mosses, the cells in the stem and veins of the leaves are so elongated, as almost to resemble woody and vascular structure, while the ferns possess dotted and reticulated ducts, like those of the phanerogamia, and may therefore be inferred to convey the same kind of fluid, though little has been actually observed as to the circulation of sap in this order of plants. The course of the sap in exogens is well known; but this cannot be said of endogens, though there is good reason for believing that it ascends by the ducts and woody fibre, and returns along the cellular portion of the stem. The author is of opinion that the ascent of the sap depends on two sets of causes; one the propulsive motion in the roots, by endosmose, the other the attraction upwards, occasioned by the vital processes in the leaves. The motion of the descending sap is owing essentially to the nutritive processes which it supplies, but it is promoted by gravitation and the vibrations of the stem produced by wind. The following remark has very important bearings.

"If the description given by Schultz, and confirmed by other observers, of the motion of fluid witnessed by them, really applies to this general circulation of nutritious or elaborated sap, it obviously bears a very close analogy with the movement of the blood in the capillary vessels of animals; since this also would seem less dependent upon the *vis a tergo* or impulsive force of the heart, than upon the new set of attractions and repulsions created between the particles of the fluid and the surrounding tissues, by that mutual action in which the process of nutrition consists. This *vital circulation,* as it has been termed, may be seen not only in detached parts, in which it continues for some time, but also in the growing plant." (p. 227.)

The progressive development of the circulating system in vascular plants corresponds with the gradations which we have noticed in the different orders. The embryo within the ovule absorbs from its whole surface, and therefore needs no particular channels of transmission for the nutrient fluid; but, as it lengthens in the process of germination, the fluid is conveyed as in fungi by elongated cells and intercellular passages. When the true leaves are expanded, woody or vascular structure becomes distinct, but it is curious to observe that the ducts after assuming a spiral disposition in young plants, are afterwards converted into the dotted form.

The preliminary remarks on the circulation in animals are excellent: our space, however, scarcely allows of extract; but we must quote the following observation:

"In proportion as the function of absorption is restricted to one part of the surface, that of respiration will be limited to another; and the processes of nutrition, and the formation of secretions will go on in parts of the structure distant from both; and all these must be brought into harmony by vascular communication, the arrangement of which will evidently vary from the most simple to the most complicated form, according to the number and variety of the offices to which it is subservient." (p. 229.)

The porifera, as regards their independence of a circulation, are in the same predicament as the lowest cellular plants. The appearance of vessels in some of the infusoria described by Ehrenberg, the author is disposed to ascribe to the reticulated distribution of the digestive cavity. In the radiata we discover the first traces of a circulating apparatus, not however in the acalephæ, (the canals of which are only prolongations of the digestive cavity and convey a crude not an elaborated fluid,) but in the echinodermata. In these animals there is "a gradual restriction of the digestive cavity, to the central portion of the structure." Thus, in the asterias, a vessel lies on the surface of each alimentary canal and is connected by minute branches with the cæca. The vessels from the rays join with those of the stomach, and form a ring round the upper part of the body, and this circular vessel is connected with a similar ring, surrounding the anus, by descending branches. The echinus possesses a contractile cavity, the first trace of a heart, near the anal termination of the intestine; but in the holothuria a pulsating vessel accompanies the intestine, analogous to that of the articulata. In this class, the principal sources of impulse to the circulating fluid are the active capillary processes of nutrition and respiration, aided by the muscular movements of these animals. The respiration being all but universal in insects, from the numerous tracheæ, there is but little distance to be traversed between these parts and the tissues. In the mollusca, on the other hand, a concentrated organ of impulsion is required by the insulation of the respiratory apparatus, and by the torpid habits of this class. We shall not follow the author in his very clear and exact description of the modification of the circulation in the upper classes; but it gives us much satisfaction to notice that, although allowing that in animals possessing a muscular heart the *vis a tergo* is the *chief* agent in the circulation, he yet maintains that the nutritive and secretory changes in the tissues (which may be expressed by the term *capillary power*, provided we do not imply a contraction of the capillary vessels,) have still some share in maintaining the motion of the blood in the vessels, and certainly modify in a great degree the quantity supplied to different organs. This capillary power which is the only source of motion in the lowest plants and animals, becomes gradually subordinated, as we ascend the scale, to the central impulsion, but is never entirely abolished. This position is, we think, rendered perfectly impregnable by the facts with which the author surrounds it. To state them in brief:—The continuance of the blood's motion in the capillaries of cold-blooded animals, after excision of the heart—the emptying of the arteries after slow natural death—the continuance of secretion after the cessation of the heart's action—the irregularities in the capillary currents, and their unequal velocity, as observed under the microscope—the increased determination of blood or *turgor vitalis* in organs which have a temporary increase of function—the augmented supply in morbid conditions of parts though the heart is not pulsating more forcibly than natural—the arrest of the pulmonary circulation in asphyxia, prior to the cessation of the *vis a tergo*—the movement of red blood in the embryo before any pulsating vessel can be seen (a fact proved by Von Bär, though doubted by Dr. Allen Thomson, and others,)—the movement of red globules in coagulable lymph before any communication with adjoining vessels—and, lastly, the circulation in acardiac fœtuses, of which a memorable example has been described by Dr. Houston.* Of this last instance, it is observed, "From a careful examination of the vascular system, it appeared impossible that the heart of the twin fœtus could have caused the movement of the blood in the imperfect one; and this must, therefore, have been entirely similar to the circulation of elaborated sap in plants, being maintained by the nutritive changes occurring in the capillaries; an effect not the less certain because we are as yet unable to explain it satisfactorily." (p. 250.)

The embryonic development of the circulating system successively imaging, in a single individual of the higher classes, the permanent states in the inferior gradations, is treated with a degree of fulness, proportionate to the importance of a subject which reflects so much lustre on modern science.

The chapter concludes with a concise account of the principal malformations of the heart and the aorta in the human subject.

We must reluctantly pass over the intermediate chapters, in order that we may glance at the author's views of Reproduction in Vegetables; views, which bear the stamp no less of truth than originality. The general considerations respecting this function are well worthy of attention, more especially as some remarks on the limits within which the doctrine of *equivocal*, as distinguished from *spontaneous* generation may be rationally maintained.

Reproduction, like absorption, respiration, &c. is performed by every part of the simplest plants, but in the higher orders we find it restricted to a special organ. Of the former, the Protococcus nivalis is a remarkable example. Each vesicle of this plant contains granules, susceptible of development into cells resembling the parent vesicles. These, after bursting and thus destroying their envelope, escape from it, move spontaneously in the water, and also generate within themselves new cells which are in like manner burst by the embryo within them, and are disorganized. Thus the whole of the structure is concerned in reproduction.

"The same process will be found to take place in the highest plants, with this difference,—that as the whole system is not concerned in the formation of the embryo, but only a very small portion of it, that portion alone ceases to exist as soon as its function is performed, the life of the parent remaining uninjured. In the higher cryptogamia, the reproductive cells, containing the germs, are distinct from the rest of the structure, and are developed only from a particular part of it; they are denominated *spores*. And in the phanerogamia this is also the case, the reproductive cells being there termed *pollen*; but an additional organ is here developed, for the purpose

* Communicated to the British Association in 1836. See our 2d Vol. p. 596, or Dub. Med. Journ. 1837.

of receiving and nourishing the embryo on its first liberation, and of thus enabling it to advance ultimately to a more exalted condition than it could have attained if left to its own resources from the beginning. In all instances the reproductive cells have essentially the same character. They contain an immense number of minute granules, swimming in fluid, and endowed with a peculiar spontaneous motion, which may be observed both before and after their liberation." (p. 397.)

In the higher algæ, the function is somewhat more insulated; some of the cells, which by their union constitute an individual, containing no granules, others evolving them abundantly. The germs after escaping by pores, as in the confervæ, move actively about. But in the fucoideæ, they are more inert and are conveyed by gravitation to the places fit for their development. In some of this class, a particular portion of the surface is devoted to the formation of the granules. Lichens are propagated by much the same method, excepting that in some of them the process is more similar to *germination.* But of the fungi the whole structure seems devoted to the reproductive system. This system in the *mosses and ferns* is represented by spore-cases or thecæ, which occupy a smaller and smaller portion of the plant, the higher we ascend the scale. The spore is the only organ of generation in these vegetables. The capability of producing a germ which may develop itself into a new plant, seems to be an essential property of the cells which have been designated as reproductive, just as the power of developing additional vesicles, which may remain parts of the same organism, is an attribute of those which belong to the nutritive system. The spore is then shown to correspond to the pollen-grain. Each contains the green germs or granules floating in a liquid, and each consists of two coats, the inner being very delicate. In the development of the spore the outer coat splits, and the inner is protruded in the form of a tube containing the granules; insulated portions of this tube are capable of reproduction. Into the account of the further evolution we have not space to follow the author. In the marsillaceæ, intermediate to the ferns and phanerogamia, we first meet with an *ovule,* which is a receptacle containing nutriment for the spore. In the marsilea the spore-cases, analogous to *anthers,* are inclosed with the ovules in a common envelope, and there is a direct communication between them. Neither of these parts can generate independently of the other. The essential organ in phanerogamia, as in cryptogamia, is the part which produces cells containing germs, and which is called the *anther,* the pollen-grain of which is analogous to the spore. The pistil is nothing more than a tube which conveys the germs, liberated from the pollen, to the ovules at its base; where they receive a special nutriment prepared for them and which is essential to their evolution: in this respect differing from the spores of cryptogamia, which depend on the nutriment found in the surrounding medium. The correspondence between the changes in the spore and in the pollen-grain is made out in a very interesting and satisfactory manner; and not less so between the early development of the embryo of the phanerogamia within the ovule and the evolution of the germs of cryptogamia; as well as the "general analogy between its transitory condition at different epochs and those which are permanent in the lower grades." The following is the concluding paragraph of this interesting section.

"In tracing the progressive evolution of the special reproductive apparatus in plants, we observe that although it is gradually separated from the nutritive system, in proportion as we ascend the scale, it is never entirely disconnected with it. It was formerly stated that all the parts of the flower may be regarded as metamorphosed leaves; or, more correctly, as metamorphosed forms of the elements of which leaves are the types. Even the stamens and carpels are proved, by the frequent occurrence of monstrosities, to have this character. The former often present the appearance of leaflets thickened at their edges by the formation of pollen; and these reproductive vesicles are themselves found, by observation of their early development, to differ but little in essential character or mode of production from any other form of cellular tissue. The carpels, moreover, are proved to be leaves, not only by such monstrosities as the one formerly mentioned, but by the fact of their bearing *ovules* at their edges; for these ovules are essentially *buds*, (as may be seen in particular abnormal instances), like those developed from the edges of various leaves, such as those of the *Malaxis paludosa* (Bog-orchis), and *Bryum colycinum* (one of the air-plants of the tropics), which are capable of developing themselves either separately or while still attached to the parent structure. The special reproductive organs of the cryptogamia might probably be reduced to similar elements, if their monstrosities were observed; thus, the *sori* of the ferns have been seen to be replaced by clusters of leaflets, each of them representing a metamorphosed theca." (p. 403.)

We are glad to observe, that it is the author's intention to publish his views on vegetable reproduction more at large in a separate work.

The section on Generation in animals, presents very striking analogies between the function in this kingdom and that in vegetables. But the author has been rather shackled in his illustrations, by his laudable desire of handling the subject in such fashion as need not offend the delicacy of general readers, for whose instruction he wishes to cater as well as for the professional student.

We give a brief extract from the first paragraph:

"In the *gemmiparous* propagation observed in many of the polypes, the new being is obviously nothing but an increased development of a part of the parent structure, and exactly corresponds with the bud of a plant; a similar mode of increase seems to exist in some of the simpler entozoa, where the young sprout from the interior of the cavity of the parent, and swim about, after their separation, in its contained fluid. The *fissiparous* generation, as it is called, is evidently but another form of the same plan; the parent structure not putting out a smaller and younger bud, but dividing itself into parts of which each has the power of reproducing the whole. It is among the infusoria that this mode is most characteristically seen. Thus, the Paramœcium divides itself transversely, the division at first appearing like a notch, and gradually extending itself across the body, until the halves are completely separated. Some species of Vorticellæ divide themselves longitudinally in like manner; and instances still more curious might be mentioned. Amongst many higher animals this mode of increase is practised, as already stated; but it is seldom that a more special reproductive apparatus is not also developed. The object of this apparatus, in animals as in plants, is to form and mature a germ, which, from the time of its first organization, is destined to be the rudiment or embryo of a new being, and which is separated from its parent, in the first instance at least, in a form altogether dissimilar to that which it is ultimately to assume." (p. 403.)

We had purposed extracting Mr. C.'s observations on the nervous system of the articulata, but we must be content with directing particular attention to them. Enough has, we trust, been said to induce the reader to take the earliest opportunity of carving for himself at the rich entertainment which this book affords. For ourselves, we cannot express the satisfaction we derive from the method of studying physiology, which has arisen out of the comparative anatomy and natural history of the present age, and which was never more fully and faithfully worked out than in the present volume; a happy exchange, indeed, for the old system, on the one hand, of speculating "*de usu partium*," and presumptuously placing a human mind in the position of the Creator, so as by its precognition of the final cause, to dogmatize at once on the quality and order of the phenomena presented to its observation; and, on the other hand, for the too prevalent practice of putting Nature *to the question*, or at least compelling her to betray the secrets of her agency in the groans and writhings of the very organisms on whose happiness she has bestowed such a prodigality of resources and such exquisite artifice. We infinitely prefer listening to the philosophers who repeat the beautiful tale which nature has *spontaneously* whispered to them; for our kind mother shows not reserve towards those who in a due spirit of humility have enquired at first respecting the simpler of her works, and thus gradually prepared themselves for comprehending her more marvellous and complex operations.

The literary execution of this treatise, is on a par with its scientific merits. The style is adapted to the subject, simple, vigorous, and translucent. Ornament would be misplaced; but the language occasionally rises into eloquence. The author has adopted the use of numbered paragraphs; a plan which has many and great advantages, for the purpose of reference, but which is not altogether unattended with inconvenience. That which most strikes us, is the temptation which it affords, of trusting too much to the paragraphic divisions of the argument, and not marking them sufficiently by those particles and connecting phrases which so much aid its comprehension in minds not familiar with the subject.

Six plates are appended, full to overflowing with illustrations, executed in a manner no less creditable to the artists, than useful to the student; and we are glad to see that great pains have been taken with the explanation of the individual figures. An elaborate index closes the volume, a most important feature in a work like this, but not always regarded by authors.

On the whole, we must be allowed to say that no treatise on physiology which has hitherto appeared in our language exceeds the present, either in the comprehensiveness of its principles or in the value and abundance of its facts. Of many of the views, the author may fairly and proudly claim the paternity; but where he has adopted the opinions of others, it is plain that he has thought out the subject for himself, not passively received the ideas. The work is unquestionably unique in plan; and, while we have in no part found it lagging behind the most recent steps of modern science, it has in many instances appeared quite in advance of it. Had we seen the book in manuscript, our *imprimatur* would have been inscribed, not in its usually permissive, but in its absolutely imperative sense. After this, it is hardly necessary to recommend it formally to all our readers, and to men of science of every description. The volume is dedicated to Sir John Herschel; and we know not, in the whole range of modern medical literature, a production more worthy of his notice, or more truly conceived in the high and philosophical spirit which distinguishes his own writings, and has added fresh lustre to a name already immortal.

## Figures and Tables

**Figure f1:**